# Reconstruction of Optical Properties in Turbid Media: Omitting the Need of the Collimated Transmission for an Integrating Sphere Setup

**DOI:** 10.3390/s24154807

**Published:** 2024-07-24

**Authors:** Dongqin Ni, Niklas Karmann, Martin Hohmann

**Affiliations:** 1 Institute of Photonic Technologies (LPT), Friedrich-Alexander-Universität Erlangen-Nürnberg (FAU), Konrad-Zuse-Straße 3/5, 91052 Erlangen, Germany; dongqin.ni@fau.de (D.N.);; 2 Erlangen Graduate School in Advanced Optical Technologies (SAOT), Paul-Gordon-Straße 6, 91052 Erlangen, Germany

**Keywords:** optical properties, machine learning, spectroscopy

## Abstract

Currently, the most reliable approach to reconstruct optical properties, namely absorption coefficient, reduced scattering coefficient, scattering coefficient and asymmetry factor, of turbid media is through inverse Monte Carlo simulation. To determine these optical properties, three measurements are required: total transmission, total reflection and collimated transmission. However, the accurate determination of the collimated transmission is very difficult. To overcome the difficulty of measuring the collimated transmission, it is proposed to measure the total transmission and total reflection of the same sample with two different thicknesses instead. To prove this alternative solution, machine learning is used to prove that the repeated measurement for two different thicknesses carries all the necessary information. As a result, all four optical properties can be measured with high accuracy, particularly for interpolation problems where the test data fall within the range of the training data. For extrapolation problems, high accuracy can be achieved in the determination of at least the absorption coefficient, reduced scattering coefficient and scattering coefficient. Hence, these results allow that in the future, an easier and therefore more precise reconstruction of the optical properties is possible, potentially even with inverse Monte Carlo simulations as the current standard.

## 1. Introduction

Various techniques have been accessible for several decades to ascertain optical properties, namely absorption coefficient ($\mu_{a}$), reduced scattering coefficient ($\mu_{s}^{\prime }$), scattering coefficient ($\mu_{s}$) and asymmetry factor (*g*) [1]. These techniques can be classified into direct and indirect methods [2]. Generally, direct techniques necessitate the exact cutting of tissue and the use of thin samples, although recently, some studies have suggested that these restrictions have been eased [3]. Nevertheless, indirect methods such as Kubelka–Munk (KM) [4], inverse adding doubling (IAD) [5,6], diffusion approximation (DA) [7] and inverse Monte Carlo simulations (IMCS) [8,9] remain the most widely used techniques to date. While the first three methods were more commonly used in the past, they have a disadvantage: either requiring media where light cannot escape sideways or only functioning properly over limited optical properties. They have been the preferred choice over IMCS because of their higher speed of computation. Despite these disadvantages, the significant increase in computing power has now overcome the shortcomings of IMCS.

In spatially resolved setups, IMCS is still considered too slow, as per pixel per wavelength at least $10^{6}$ photons are required to reduce the noise strong enough for the optimiser. Therefore, indirect calculations are sometimes replaced and/or supported by look-up tables or machine learning (ML). ML in particular can be used to derive OPs under various conditions. For instance, Jäger et al. [10] reconstructed the absorption coefficient and the reduced scattering coefficient from the spatially resolved reflectance. The same can be done for diffuse reflectance spectroscopy techniques [11,12], spatial frequency domain imaging [13], spatio-temporal response [14] and more.

Despite these advances, for the accurate reconstruction of OPs, integration spheres are used to determine total transmission (TT) and total reflection (TR), as they allow the accurate measurement of TT and TR for the reconstruction of the absorption coefficient and reduced scattering coefficient [15,16]. Nevertheless, to separate $\mu_{s}$ or *g* from the reduced scattering coefficient $\mu_{s}^{\prime }=\mu_{s}\cdot \left(1-g\right)$ for ex vivo applications, it is usually necessary to measure the collimated transmission (CT). Measuring CT is a daunting task, particularly for optically thicker samples, despite the recent advancements in methodology [3,17]. Alternatively, it was demonstrated that the application of random lasing offers a direct pathway to access the scattering coefficient [18]. Achieving significant progress for general use is possible if all three key optical properties can be reconstructed without relying on CT. As an example, Sun et al. [19] replaced TT and TR with spatially resolved reflectance measurements. This approach allowed them to prove that modifying the measurement system using a simulation-based metamodel design can provide access to the desired optical properties.

Although Sun et al. [19] demonstrated that TT and TR can be substituted, their study only reconstructed the reduced scattering coefficient. To derive the scattering coefficient and asymmetry factor for integrating sphere measurements rather than the reduced scattering coefficient, it is necessary to measure TT, TR and CT. It has already been shown that ML provides excellent results for estimating optical properties using integrating sphere measurements with the usage of CT [20]. However, as previously stated, it is advantageous to avoid measuring CT. Therefore, one potential solution is to substitute the measurement of CT with a second measurement of TT and TR, but using a different sample thickness. Thus, this study aims to assess whether the measurements of TT and TR for two different sample thicknesses are adequate for computing $\mu_{a}$, $\mu_{s}^{\prime }$, $\mu_{s}$, and *g*. Machine learning is used for the assessment due to its expected effectiveness and ease of use.

Since ML seeks to establish the relationship between two data sets, samples of known OPs are measured and the mapping between the measured TT and TR to the OPs is evaluated. Additionally, to accommodate measurements with varying *g*, the samples are measured within a specific spectral range, as *g* is wavelength-dependent. Therefore, the spectral information has been omitted in this paper to allow for variations in the asymmetry factor, which would otherwise be challenging to access.

## 2. Methods

The methodology section is divided into three parts. The initial part explains the experimental setup. In the succeeding part, the samples are presented in detail and the range of optical properties is shown. The third part explains the ML method.

### 2.1. Set-Up

**Figure 1:**
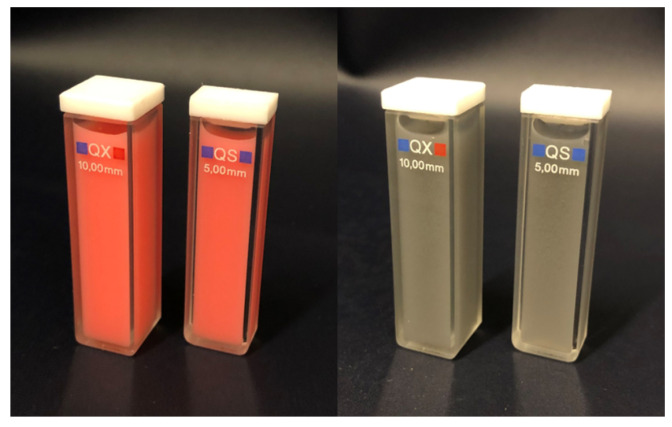
Example of filled cuvettes with IL and red ink (**left**) and black ink (**right**).

The TT and TR measurements are conducted using a commercially available spectrophotometer (Shimadzu UV-3600 UV-VIS-NIR Spectrophotometer, Double Beam, Three Detector System, Kyoto, Japan) equipped with the LISR-3100 Integrating Sphere (IS) attachment. The entrance port for the TT measurement is a square with a size of 14 × 14 mm^2^ and the input beam is also squared with a dimension of 8 × 8 mm^2^. The entrance port for TR is a rectangle with a size of 14 × 30 mm^2^ and the input beam is also rectangular with a dimension of 8 × 25 mm^2^. Two cuvettes are used for measurement; one of 5 mm sample thickness and the other of 10 mm sample thickness. The samples are illustrated in Figure 1. The choice of the sample thickness was arbitrary. It was based on the availability of the cuvettes. However, it is unlikely that other sample thicknesses would yield different results.

For each measurement, the sample is prepared by mixing ink, deionised water and IL in the appropriate concentrations with pipettes. The sample solution is then filled into both cuvettes. Subsequently, a cuvette is placed in front of the entrance or exit port to measure TT or TR, respectively. Both cuvettes with the different sample thicknesses are measured sequentially. The spectral range of the measurement is 500–800 nm with a resolution of 2 nm. The upper limit is determined by the high noise level of the spectrophotometer beyond 800 nm. The lower limit is chosen because the optical properties are reported in the literature only for 500 nm and above. It is typical that optical properties vary with wavelength; for example, the scattering coefficient decreases at longer wavelengths. Consequently, by measuring a broader spectral range, a greater variety of optical properties with the corresponding TT and TR can be produced, without the necessity of employing additional phantoms.

Additionally, an IS configuration introduces systematic measurement errors that should be taken into account [15]. Furthermore, it has been demonstrated that the IS measurements are contingent on the angle of incidence of the light [21]. Although this factor is rather simple for similar samples, it varies considerably when measuring samples with different scattering directions. As this study employs a diverse range of scattering strengths, the distribution of scattering angles may not be constant. This may result in a systematic error when measuring the parameters of TT and TR. It is not possible to entirely compensate for this error using the reference beam. These factors can potentially lower the accuracy of the applied machine learning techniques.

### 2.2. Sample

The samples utilised in this study were liquid phantoms composed of intralipid (IL) from Fresenius Kabi and either red ink (Modena Red from MONTBLANC) or black ink (Indian Ink from Winsor & Newton). The concentration of the ink results in a change in the absorption coefficient, whereas the IL concentration alters the scattering represented by $\mu_{s}$ and $\mu_{s}^{\prime }$.

The optical properties of the IL were obtained from the study by Aernouts et al. [22]. The ink had previously been characterised through measuring the TT with distinct ink concentrations in the absence of a scatterer in water, as it is assumed that the absorption of the ink dominates the scattering. The measurements were conducted by measuring different concentrations in order to derive the extinction coefficient by fitting the logarithmic TT with a linear function. The slope of this function is the extinction coefficient. Every absorption coefficient can be calculated by multiplying the ink concentration with the extinction coefficient. As the used concentrations are very low, there are no concentration-dependent effects. The precision of the ink characterisation is evaluated by the $R^{2}$ value of the fits. The $R^{2}$ value for the characterisation was greater than 0.995 in all cases, indicating that both the measurement process and the generation of the appropriate concentrations were accurate.

**Figure 2:**
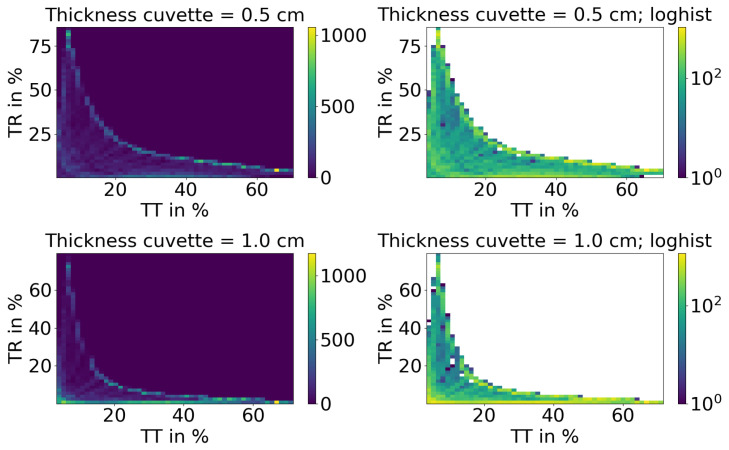
Histogram of the measured TT and TR for each of the two cuvettes. The x-axis represents TT and the y-axis represents TR. The left histograms have a linear scaling and the right ones a logarithmic scaling.

**Figure 3:**
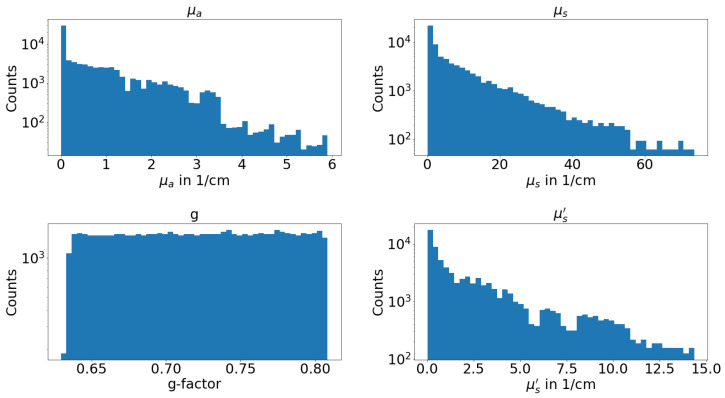
Logarithmic histogram of the four optical properties that are used as data for the machine learning.

The ink and IL concentrations used are displayed in Table 1. The ink and IL concentrations were selected to ensure that the logarithmic concentrations occupy roughly the same proportion of the space. The maximum factor between two neighbouring concentrations is two. For lower concentrations, larger relative differences were deemed acceptable, as even a large change does not lead to significant changes in TT and TR. This can be observed in Figure 2, where for large values of TT+TR, there are relatively more values. Additionally, the spectral range with higher absorption, leading to low TT and TR, is increased. This was done deliberately in order to enhance the number of available values for large scattering and absorption, which are relatively less common. The lower amount of larger scattering and absorption values can be observed in Figure 3. All phantoms consist of IL and one ink type. TT and TR are measured once for every ink concentration at a specific IL concentration. This implies that the identical scattering coefficient is incorporated into the data set thirty-one times for each wavelength. During each occurrence of various ink or ink concentration, a distinct absorption coefficient emerges. This is the reason why the TT and TR measurements manifest variations. There are a total of 496 measured phantoms, each having 151 spectral data points. This amounts to a total of 74,896 data points available.

Each set of measured reflectances and transmittances, together with their corresponding optical properties, is treated as a single data point without any spectral information. This results in an increase in the number of data points for both TT/TR and OPs. The histogram of the resulting TT and TR for both cuvettes is shown in Figure 2. The top row shows the 5 mm cuvette and the lower row the 10 mm cuvette. On the left side, the histogram is linear and, on the right side, it is logarithmic. The colour represents the amount of samples for a given set of TT and TR. In a case where there is no absorption in an infinitely large medium with no Fresnel reflection, the histogram would be filled with a line with the following condition TT + TR = 100%. However, Fresnel-reflected light, as well as scattered light that may escape from the side of the cuvette, limits the maximum sum of TT + TR because this light cannot be detected. Therefore, the total sum of TT and TR is always less than 100%, as shown in Figure 2. In addition, the greater the thickness of the cuvette, the more light escapes from its sides. Consequently, a smaller area is covered for the 1 cm thick cell. Taking absorption into account, the remaining histogram is covered with data points.

Figure 3 illustrates the histogram of the assessed optical properties. The entire range is covered, with a focus on the lower values. Accordingly, better results are expected for lower values using the ML. The highest absorption coefficient is lower than the highest reduced scattering coefficient. As the absorption coefficient has a greater effect on TT than on TR, greater absorption will result in a greater reduction in TT. At higher absorption coefficients, TT would therefore be too low to be resolved by the spectrophotometer.

### 2.3. Machine Learning

**Figure 4:**
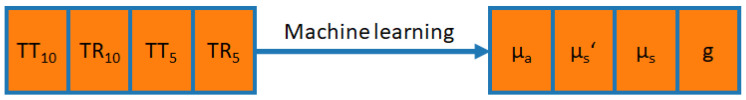
Scheme of the data sets for ML. Out of the measurements from TT and TR from both cuvettes the OPs are reconstructed.

Figure 4 illustrates the fundamental training scheme for the data. The absorption coefficient, reduced scattering coefficient, scattering coefficient and asymmetry parameter are derived from two measurements taken from TT and TR for two sample thicknesses. For machine learning, three regressors are utilised, namely K-nearest neighbour (KNN), random forest (RF) and a neural network (NN). The complete data analysis is carried out in Python, whereby for KNN and RF scikit-learn [23] was used and for NN tensorflow [24] was used. KNN and RF are used with default settings as parameter optimisations did not have a significant impact on the results. KNN with Euclidean distance is applied for the 5 nearest neighbours. RF is carried out with 500 trees, squared error, no maximum depth, 2 minimum samples to split, 1 minimum sample for a leaf and no maximum number of features for a leaf or a node.

The NN comprises of nine fully connected layers, which are shown in Table 2. The number of layers, the number of neurons, the activation and the normalisation strategy were chosen to provide the best regression results. The used graphic processor unit is a NVIDIA GeForce RTX 3090. For the optimiser, adam is used. The parameters are shown in Table 3.

The neural network is optimised to minimise the one minus the Pearson correlation coefficient (PCC), as the neural network converges faster on the PCC than on the mean squared error (MSE). Moreover, the PCC approach eases the need for scaling of the data. As it does not completely omit the scaling requirements, the TT/TR as well as the OPs are centered and scaled with the command “StandardScaler” from scikit-learn. It sets the mean-value of the data to zero and sets the standard deviation to one. Nevertheless, the regression results are incorrectly scaled due the usage of the PCC. As a result, a linear function is used to fit the training and predicted training data in order to achieve correct scaling of the predictions. The same linear fitting function with parameters from the training data is applied to the test data. Apart from the NN, regressors are trained and tested one OP at a time, simplifying the training process.

**Figure 5:**
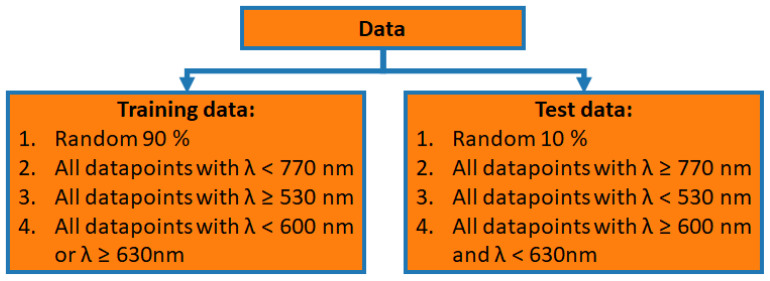
Scheme of the different training and test sets.

To conduct the analysis, the data are divided into training and test data. Four distinct arrangements are selected, as illustrated in Figure 5. Ten percent of the data is randomly chosen as test data, and the remainder is employed as training data. In this situation, the machine learning algorithm possesses identical scattering coefficients in both the training and test sets. Nevertheless, TT and TR vary because of varying absorption coefficients. Nonetheless, this can still affect the regressors. In all other scenarios, one wavelength range is designated for testing and the others for training to avoid this overlap: In the second instance, all data points that have a wavelength shorter than 770 nm are utilised for training, and longer wavelengths are utilised for testing. For the third case, data points with wavelengths below 530 nm are reserved for testing and the remainder for training. For the final case, data points ranging from 600 nm to 630 nm are used for testing, and the remaining points are used for training. As a result, there are two categories of test data generated from these data sets. For the test data with a wavelength shorter than 530 nm and the test data with a wavelength longer than 770 nm, some of the values for the scattering coefficient, the reduced scattering coefficient and the absorption coefficient are outside the range of the training data. However, this effect does not occur in the other two cases.

Additionally, the analysis of the best regressor’s error is presented in detail. Due to the amount of data, it is included as an attachment. The error for each of the four test data sets is analysed with respect to all four optical properties by evaluating the error of the scaled data used for regression. This has the advantage that the error can be compared over a wide range of OPs, including different types of OP. The error for each set of test data is calculated as the predicted OPs minus the real OPs. A histogram is then generated to display the frequency of a certain scaled error for a given OP. The final error is calculated by finding the centre of mass of the histogram, ensuring that the information is presented in a clear and logical manner.

## 3. Results and Discussion

**Figure 6:**
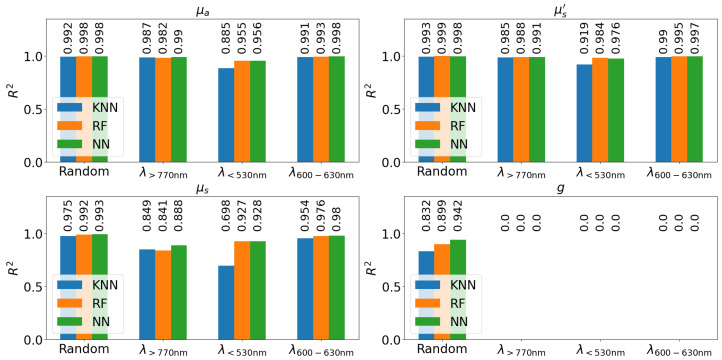
Bar plot of the $R^{2}$ of the regression for the different regressors for each OP. The x-axis shows which part of the data is the test data sets while the y-axis shows the $R^{2}$.

Figure 6 illustrates $R^{2}$, the variance in the test data that can be explained by the regression. The random test data shows that all four OPs can be accurately recovered with minimal error. For the asymmetry factor, the value of $R^{2}$ only attains 0.93. Nevertheless, all OPs can be reconstructed in this case. In the other three situations, $\mu_{a}$, $\mu_{s}^{\prime }$ and $\mu_{s}$ can be reconstructed with high accuracy. The best results are achieved when the test data are in the 600–630 nm range, since both higher and lower values are available for the training data.

In the case of absorption coefficient (Figure 6 top left), almost all scenarios demonstrated good results. As red ink results in high absorption coefficient below 530 nm, it falls outside the range of values of the training data. Consequently, in high absorption cases, $\mu_{a}$ is underestimated, which results in a lower $R^{2}$ value. As this effect does not occur in the case of test data λ>770 nm, it is inferred that this error results from an inadequate amount of training data. Adding more training data can resolve this issue.

All the test data cases show excellent results for the reduced scattering coefficient (Figure 6 top right). The results are slightly inferior when the test data have wavelengths below 530 nm. This is due to the fact that less similar training data are available as the scattering is higher for shorter wavelengths. This is caused by the absence of a similar amount of training data, as previously mentioned.

The scattering coefficient $\mu_{s}$ (as shown in Figure 6 bottom left) exhibits the same behaviour as the absorption coefficient and the reduced scattering coefficient. Sufficient similar training data produces results in very good $R^{2}$ values of 0.96 or better for test data in the random sample and within the 600–630 nm range. Increasing the random test data amount to 50% lessens the results only a little. Furthermore, with training data outside the range, $R^{2}$ remains at 0.9. The error may occur due to two reasons. First, there is insufficient training data within the spectral range of the test data and, second, the refractive index of the sample and the cuvette’s wavelength dependence alter the Fresnel reflection of light at the interfaces between air–cuvette and cuvette–sample, which in turn affects TT and TR. Therefore, the predicted value may contain errors. The effect from both is expected to be higher for the cases when test data are outside the wavelength range of the training data. The lack of training data is assumed to be the more likely explanation as the same applies to the less sensitive measurements of absorption coefficient and reduced scattering coefficient. In summary, it is assumed that the scattering coefficient can be calculated accurately and that the same limitation applies to the training data from the other OPs.

The reconstruction of the asymmetry factor (as shown in Figure 6 bottom right) is challenging. First, a sufficient amount of similar training data is required. Second, the refractive index of the sample and the cuvette’s wavelength dependence may complicate the measurement of the asymmetry factor. As the reconstruction fails in some cases, this explanation is considered more likely than in the case of the scattering coefficient. Despite this, even when 50% of the test data is randomly omitted, the $R^{2}$ drops to only 0.8. In all other situations, the asymmetry factor cannot be reconstructed.

For all OPs, the extrapolation tends to show problems with the reconstruction of OPs. In general, extrapolation beyond the training range presents a significant challenge. This is a recognised issue, particularly in the context of neural networks. Figure S9 (bottom left) in the attachment illustrates that extrapolation with the neural network in this study is not feasible, given that neural networks are inclined to refrain from providing data outside the training range. For an absorption coefficient exceeding 4.5 per cm, the error increases as the predicted value is just 4.5 per cm. However, when the training range is either larger or smaller than the extrapolation range, extrapolation works. The comparison can be found in the Figures S13–S16 in the attachment. To mitigate this extrapolation issue, it would be optimal to ensure that the training data encompass the full spectrum of potential data sets generated by the measurement device. This would represent a highly effective mitigation strategy, particularly given the demonstrated efficacy of interpolation.

## 4. Limitations

There are a few potential limitations of this study. First, the used ink is treated as pure absorber in this study despite the fact that it displays also some scattering properties [25,26]. However, the concentration of the ink has to be fairly low so that TT is high enough to be measured. Hence, the effect is expected to be small. Furthermore, the noise from the spectrophotometer has also to be taken into account. The noise in our spectrophotometer is nearly constant independent of the measured value of TT and TR. Thus, for higher absorption coefficients, the relative noise is higher, as especially TT gets lower. Therefore, it is expected that the current limitation is the noise of the measuring system and not the additional scattering from the ink.

Second, the phase function used is only approximated by the asymmetry factor, as the phase function is not entirely encapsulated in the asymmetry factor. Hence, the results might differ if the experiments are carried out with real tissue instead of intralipid. Despite these two limitations, it is expected that the results can still be generalised. However, the final $R^{2}$ might differ. Furthermore, the OPs of IL are derived from the literature and the scattering phase function is summarised by the asymmetry factor. Consequently, the actual scattering behaviour also differs from the description provided by the asymmetry factor. This effect may also influence the results presented in this paper.

Third, the trained regressors in their current state are only applicable to the geometry and refractive index of the sample and cuvettes in this study. However, this limitation can be overcome for NNs. Pre-trained NNs can be adapted to minor changes in the environment by training them within the new environment. While the NN has to learn the physics for the current geometry and refractive index, the basic physics do not change when the geometry and refractive index change. Therefore, with the training of a few new samples, the NN should be adapted. Alternatively, a simple, potentially single-layer scaling NN can be placed before or after the NN in this study to scale the results to the new situation. Furthermore, this point also includes the possibility of small errors in the results due to variations in the spectral range, as discussed in the paper. Nevertheless, it is not anticipated that a strong influence will be observed, given that variations in optical properties result in considerably larger changes in TT and TR.

The fourth point is what happens at large optical thickness for both cuvettes. It is known that the ballistic transmission and, thereby, CT goes to zero. Here, the influence of the asymmetry factor on TR and TT is quite small and, therefore, both quantities can be adequately described by the reduced scattering coefficient and the absorption coefficient. In this study, experiments were performed for a reduced scattering coefficient of up to 15 cm^−1^. However, higher optical thicknesses could not be measured because the resulting transmission would be too low for the spectrophotometer to be resolved with a reasonable noise level. However, there is no indication that the quality of the results decreases for higher optical thicknesses. Nonetheless, a future study should confirm this concept for optical thicknesses of up to a few dozen mean free paths.

The fifth point is that only a set of two different thicknesses is investigated. It is not clear that the results can be repeated for another set of thicknesses, as the scattering and absorption in a turbid medium are multiple random processes. The effect of thickness plays an important role in determining the extent of these processes. Failure to compensate for this effect in the deep learning process may result in erroneous outcomes. Furthermore, there is currently no systematic reasoning that allows us to deduce our results from the standard scattering theory. Nevertheless, the choice of thicknesses was made on the basis of availability. They are not inherently superior to other sets of thicknesses.

## 5. Conclusions

The objective of this study is to demonstrate that a measurement of the same sample with two thicknesses for TT and TR can substitute the measurement of CT for deriving the scattering coefficient and the asymmetry factor. In other words: it is possible to replace CT measurement with a second measurement of TT and TR for the same sample but with different thickness. If there are enough training data available, the reconstruction of $\mu_{a}$, $\mu_{s}$, $\mu_{s}^{\prime }$, and *g* without CT is possible in the future with high accuracy. In spite of that, for random test data, the $R^{2}$ value exceeds already 0.93 for all optical properties. Nevertheless, extending the results to data beyond the range of training data is limited. Thus, interpolation works efficiently, but extrapolation is not feasible at the current stage. In future, the current machine learning approach might be even transferred to a more formal approach or the standard inverse Monte-Carlo approach.

## Figures and Tables

**Table 1:** Used concentrations of ink and IL. All combinations of ink and IL concentrations are used for the analysis.

**IL** concentration in volume %															
0.0125	0.025	0.05	0.1	0.15	0.2	0.3	0.4	0.6	0.8	1	1.25	1.5	2	3	4
**red ink** concentration in volume %															
0.01	0.02	0.03	0.04	0.05	0.075	0.1	0.125	0.15	0.2	0.25	0.3	0.35	0.4	0.45	0.5
**black ink** concentration in volume %															
0.01	0.02	0.03	0.04	0.05	0.06	0.07	0.08	0.09	0.1	0.125	0.15	0.175	0.2	0.25	

**Table 2:** Layers for the nine fully connected layers of the used NN.

Layer Number	Number of Neurons	Activation	Normalisation
1	600	gelu	
2	600	gelu	
3	600	selu	Layer Normalisation
4	450	tanh	
5	450	relu	
6	450	gelu	
7	450	relu	
8	300	tanh	
9	4	softmax	

**Table 3:** Used parameters for the optimiser of the NN.

Parameter	Value
Batch size	16,384
Epochs	50,000
Optimiser	Adam
$\beta_{1}$	0.95
$\beta_{2}$	0.999
ϵ	$10^{-10}$

## Data Availability

The data are available upon reasonable request.

## Supplementary Materials

The following supporting information can be downloaded at: https://www.mdpi.com/article/10.3390/s24154807/s1.
