# Supplementary material for: Reconstruction of Optical Properties in Turbid Media: Omitting the Need of the Collimated Transmission for an Integrating Sphere Setup
Source: Sensors (Basel). 2024 Jul 24;24(15):4807. doi: 10.3390/s24154807 (PMC11314773; doi:10.3390/s24154807)
Supplement: Supplementary file 1 [file sensors-24-04807-s001.zip › sensors-3038105-supplementary.pdf]

Attachment: Reconstruction of optical properties in turbid media: Omitting the need of the collimated transmission for an integrating sphere setup

Dongqin Ni[1,2], Niklas Karmann[1], Martin Hohmann[1,2,*]

15th March 2024

[1] Institute of Photonic Technologies (LPT), Friedrich-Alexander-Universität Erlangen-Nürnberg (FAU), Konrad-Zuse-Straße 3/5, 91052 Erlangen, Germany
[2] Erlangen Graduate School in Advanced Optical Technologies (SAOT), Paul-Gordon-Straße 6, 91052 Erlangen, Germany
*Martin.Hohmann@FAU.de

The attachment is categorized by the four cases of test data. This section shows the error for all four OPs of the test data as a function of each OP. Each plot is a histogram displaying the histogram of how often a specific errors appears for a given value of the OP. Thereby, the error is calculated from the scaled OP to make them comparable for the whole data range. The scaled error is defined by $OP_{\text{Predicted}} - OP_{\text{real}}$, where $OP_{\text{Predicted}}$ is the value predicted from the NN and OP_{real} the value from the measurement. It is called scaled error as it used on the scaled data. To ensure the visibility of all outliers, the values of the histogram is logarithmized to the base of ten and it is represented by the colorbar. The red line represents the center of mass of the scaled error; it can be seen as the average error.

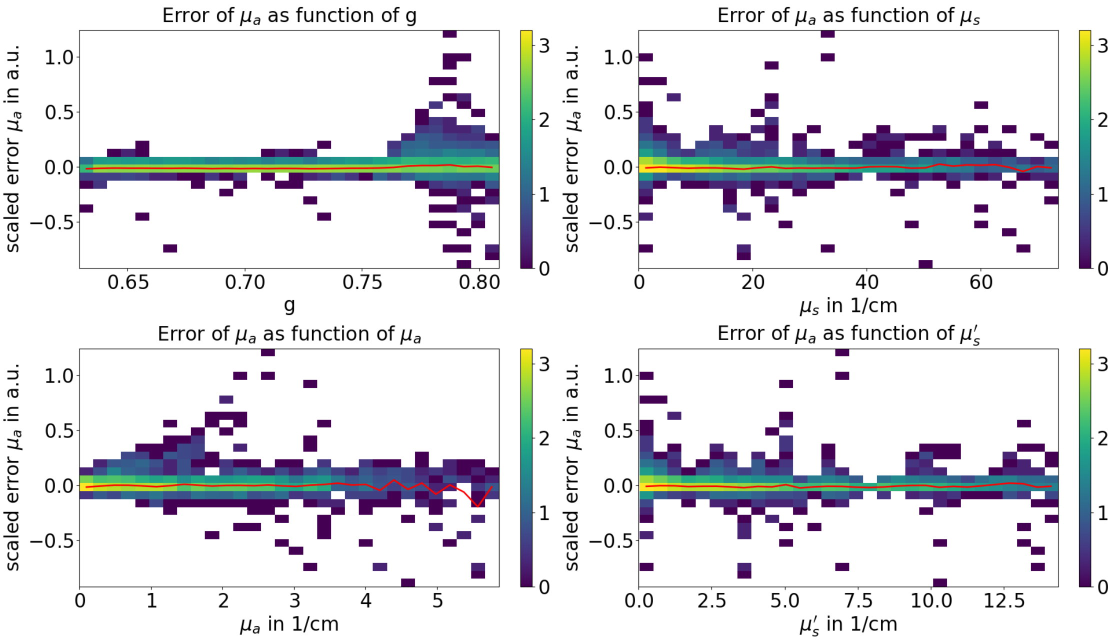

Figure S1: Scaled error of μ_a as the function of all OPs for Test data: Random 10%. The histogram is logarithmized to the base of ten and it is represented by the colorbar.

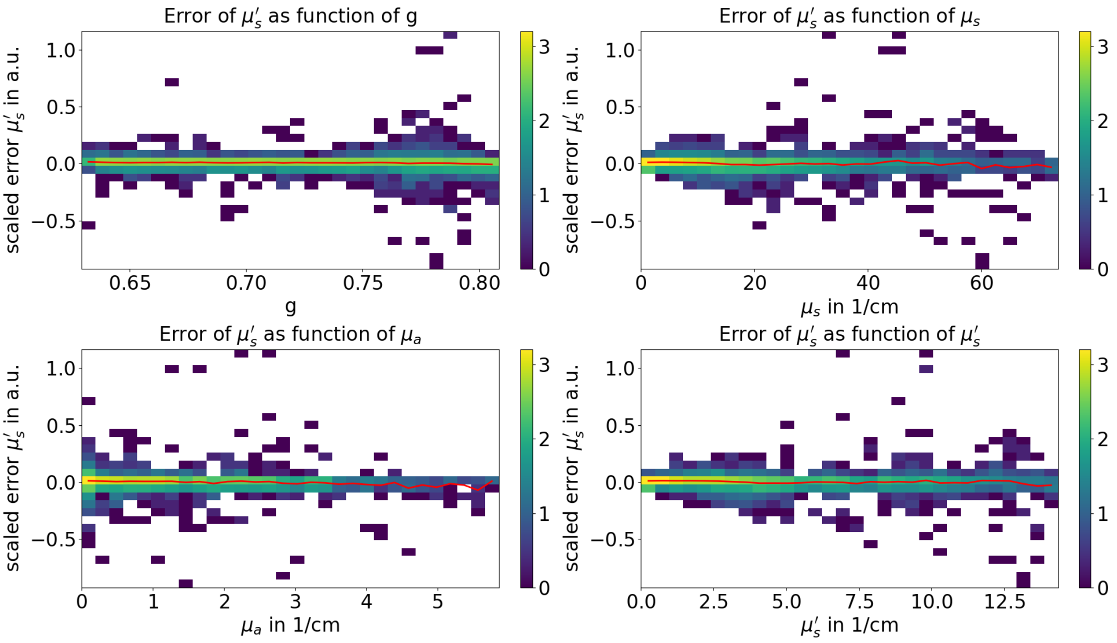

Figure S2: Scaled error of μ_s' as the function of all OPs for Test data: Random 10%.

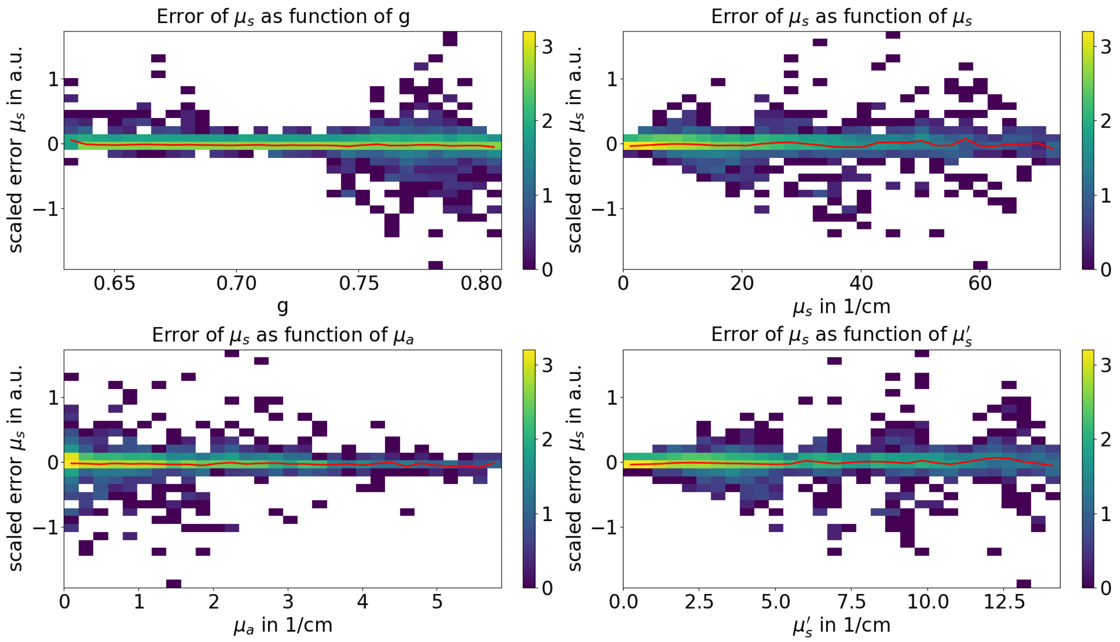

Figure S3: Scaled error of μ_s as the function of all OPs for Test data: Random 10%.

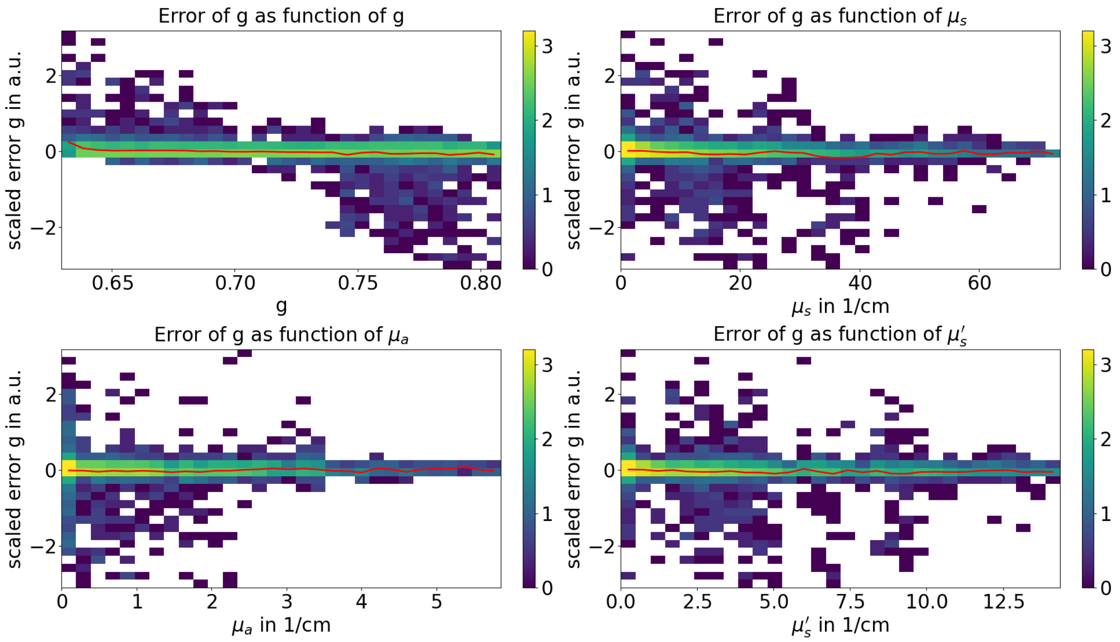

Figure S4: Scaled error of g as the function of all OPs for Test data: Random 10%.

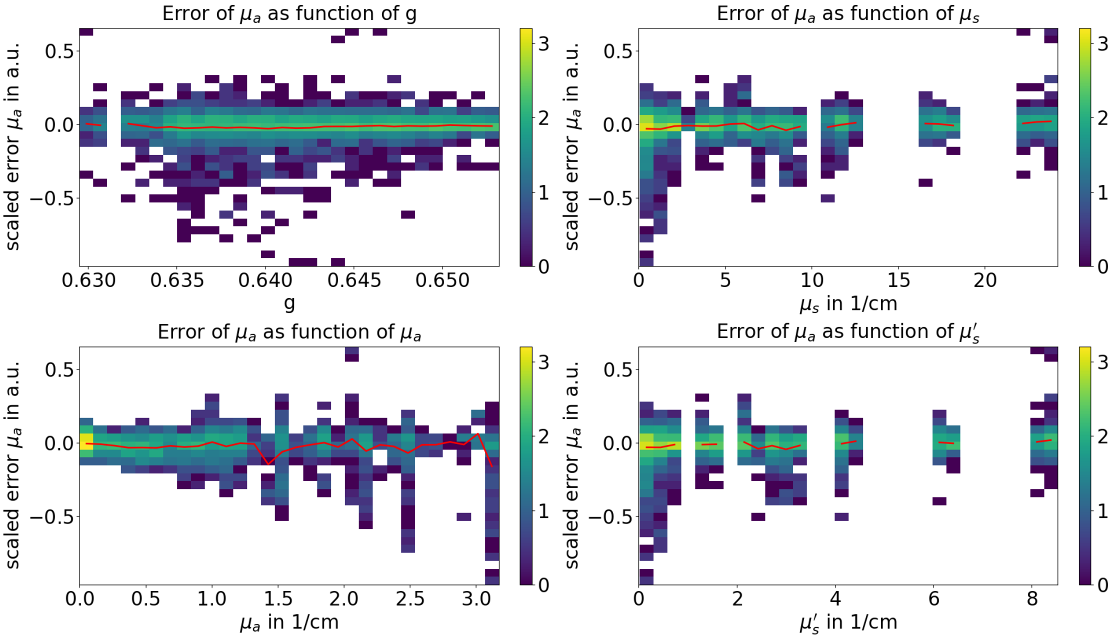

Figure S5: Scaled error of μ_a as the function of all OPs for Test data: $\lambda \geq 770$ nm.

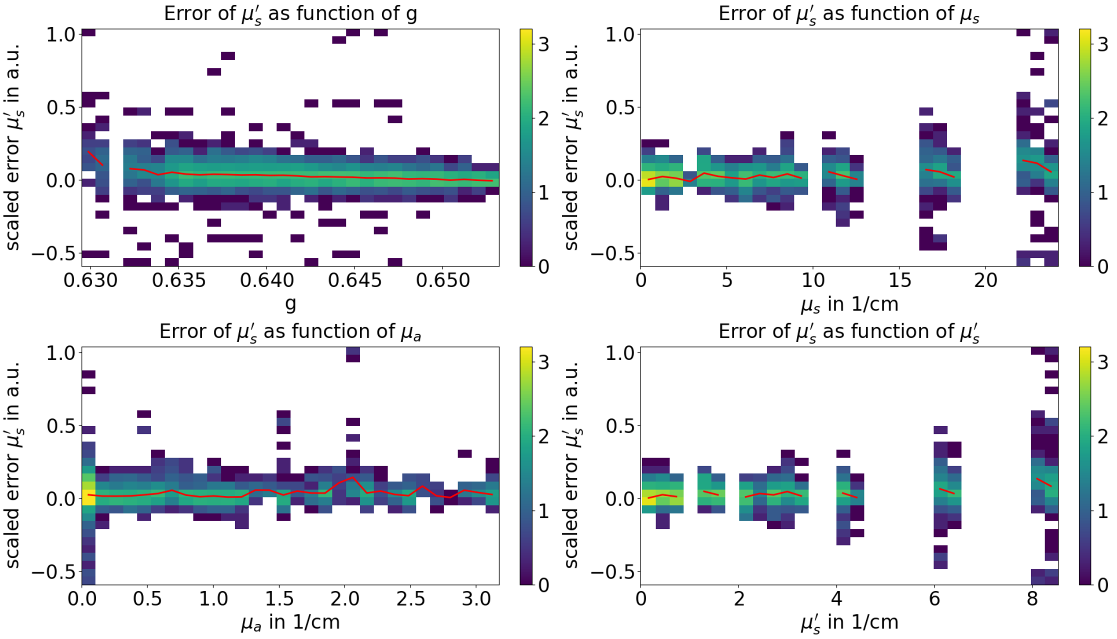

Figure S6: Scaled error of μ_s' as the function of all OPs for Test data: $\lambda \geq 770$ nm.

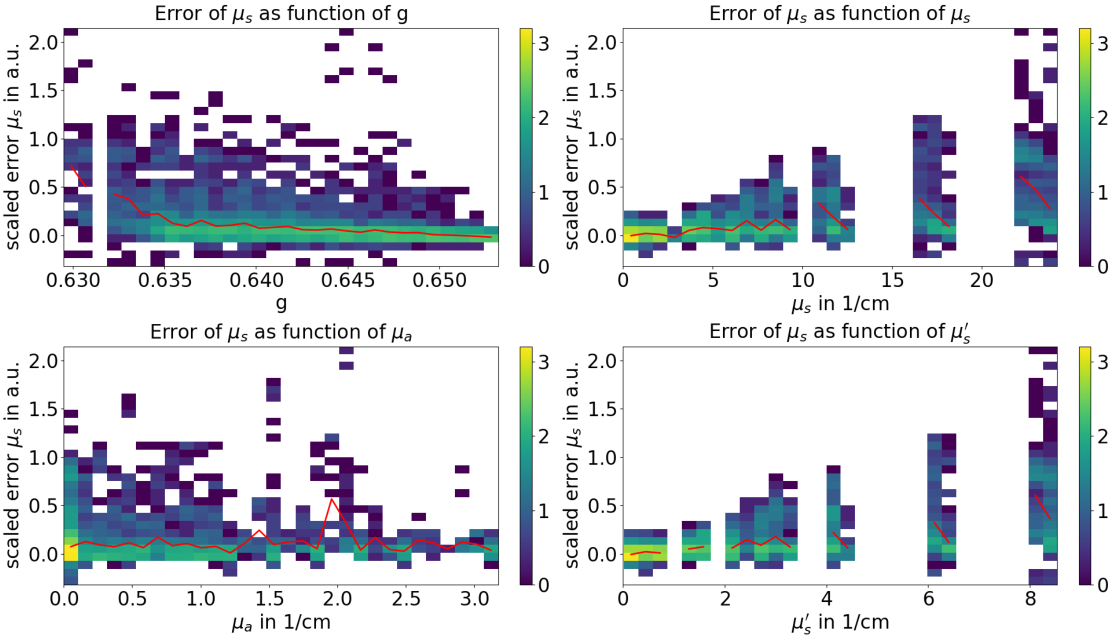

Figure S7: Scaled error of μ_s as the function of all OPs for Test data: $\lambda \geq 770$ nm.

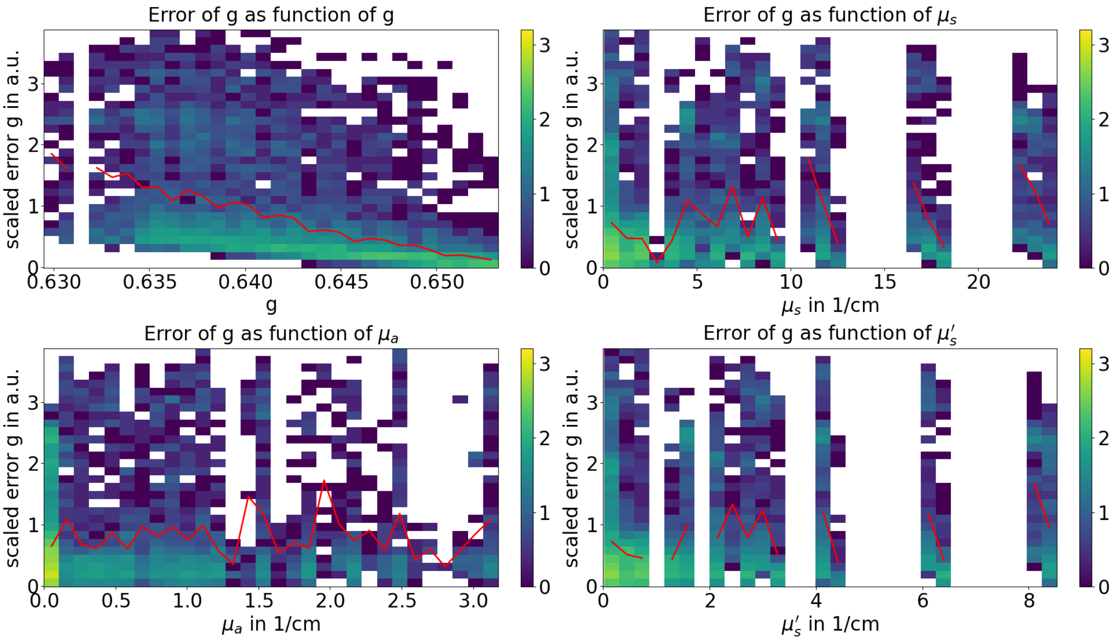

Figure S8: Scaled error of g as the function of all OPs for Test data: $\lambda \geq 770$ nm.

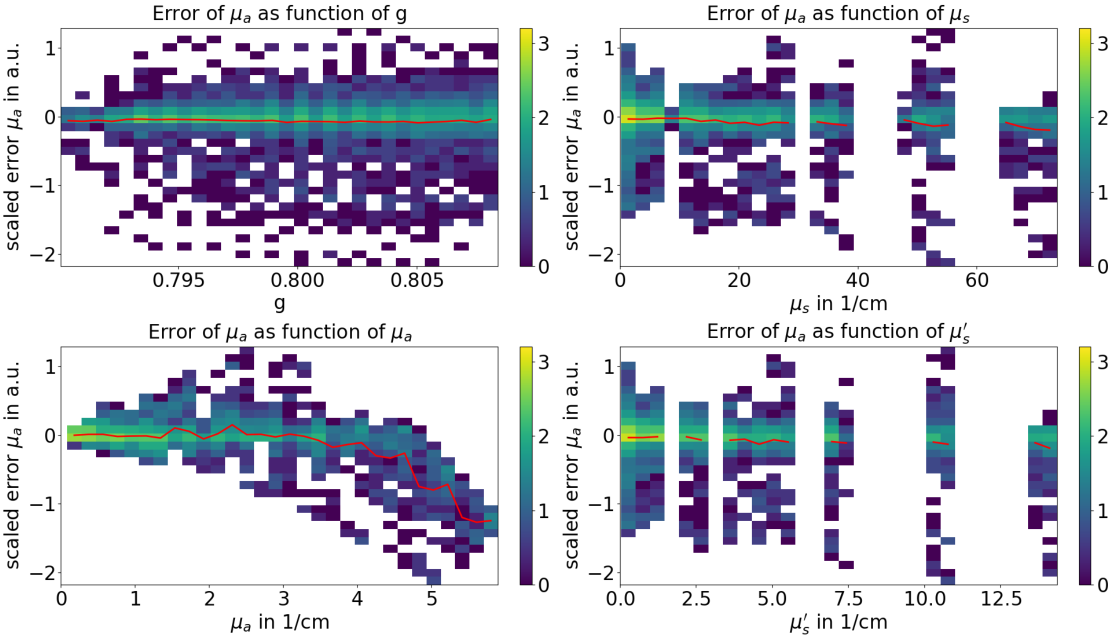

Figure S9: Scaled error of μ_a as the function of all OPs for Test data: $\lambda \leq 530$ nm.

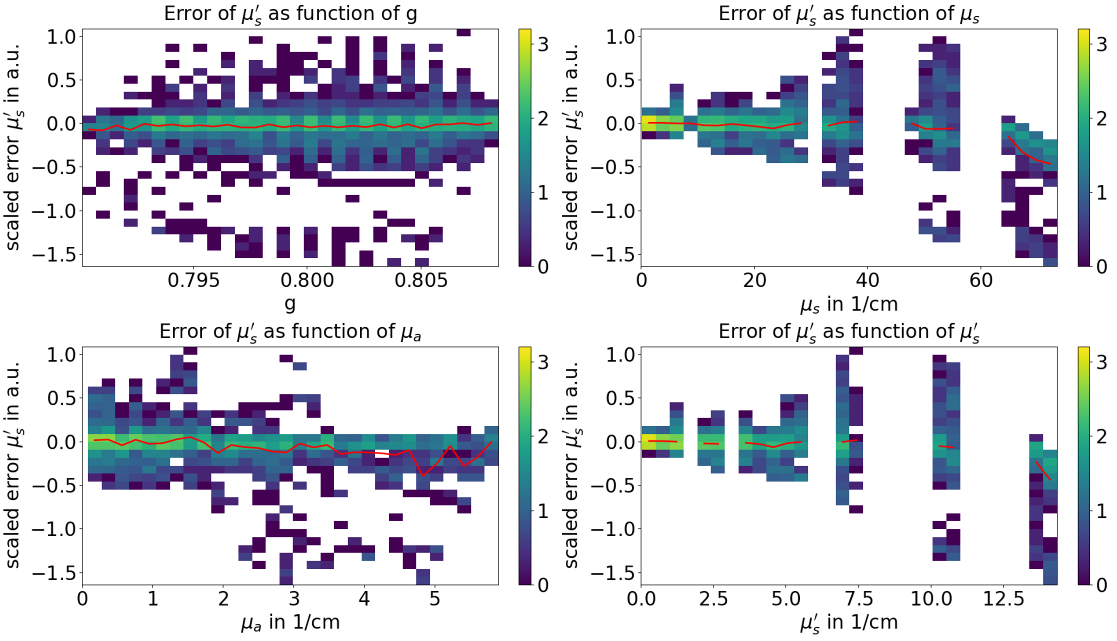

Figure S10: Scaled error of μ'_s as the function of all OPs for Test data: $\lambda \leq 530$ nm.

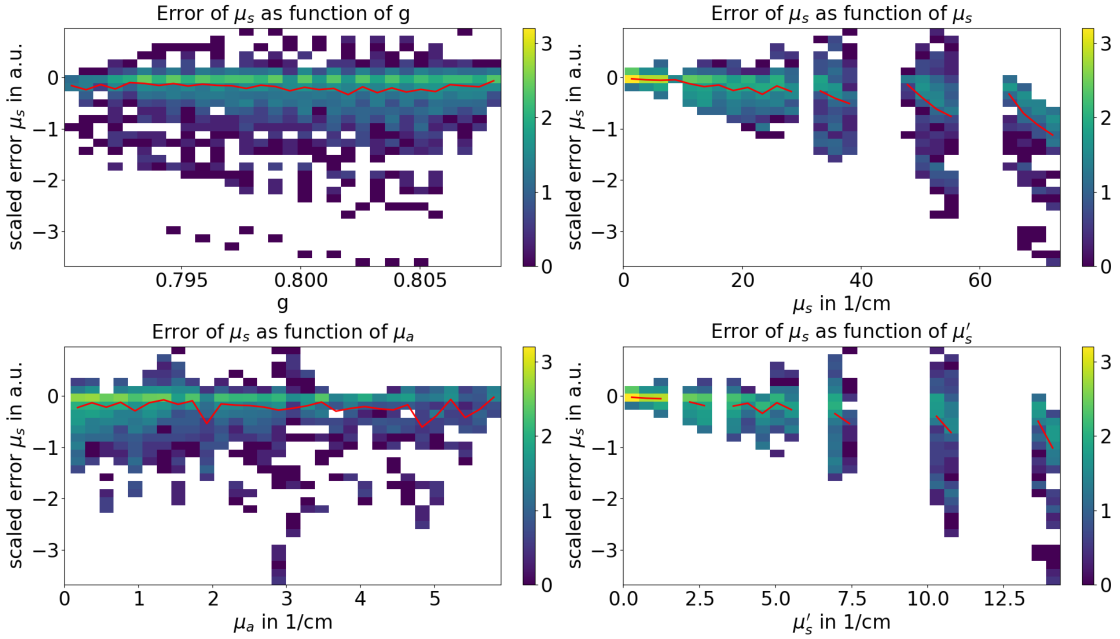

Figure S11: Scaled error of μ_s as the function of all OPs for Test data: $\lambda \leq 530$ nm.

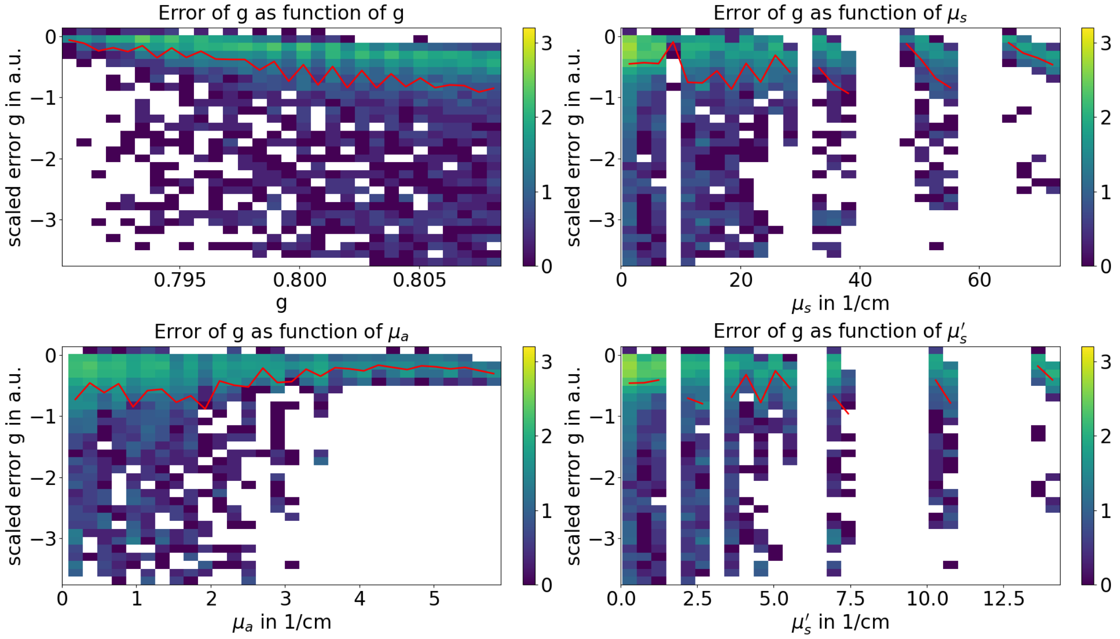

Figure S12: Scaled error of g as the function of all OPs for Test data: $\lambda \leq 530$ nm.

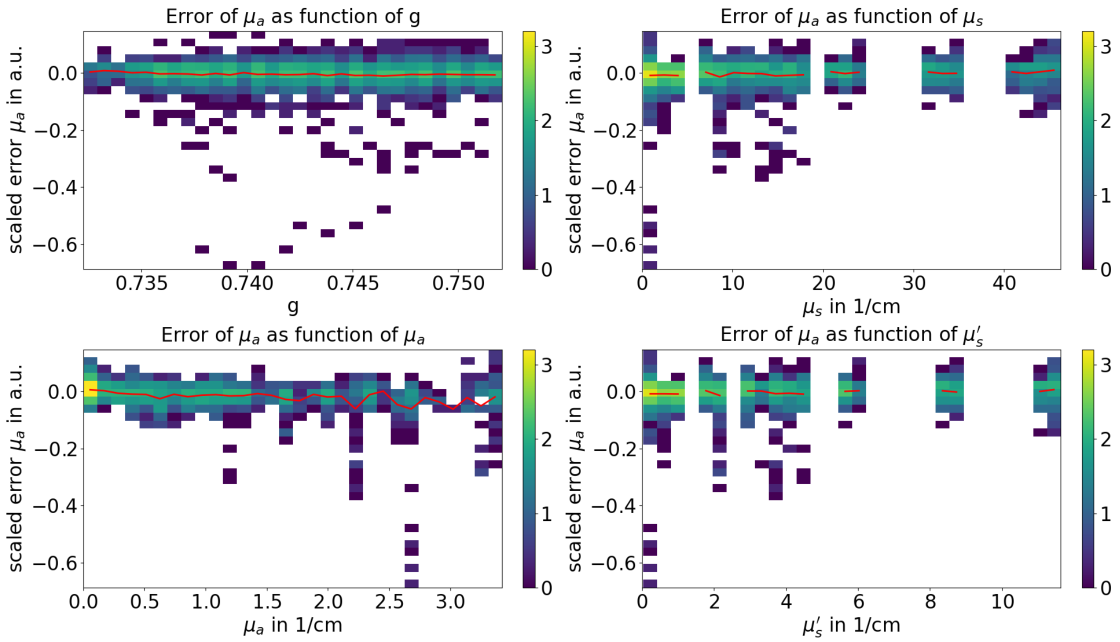

Figure S13: Scaled error of μ_a as the function of all OPs for Test data: 600 nm $\leq \lambda <$ 630 nm.

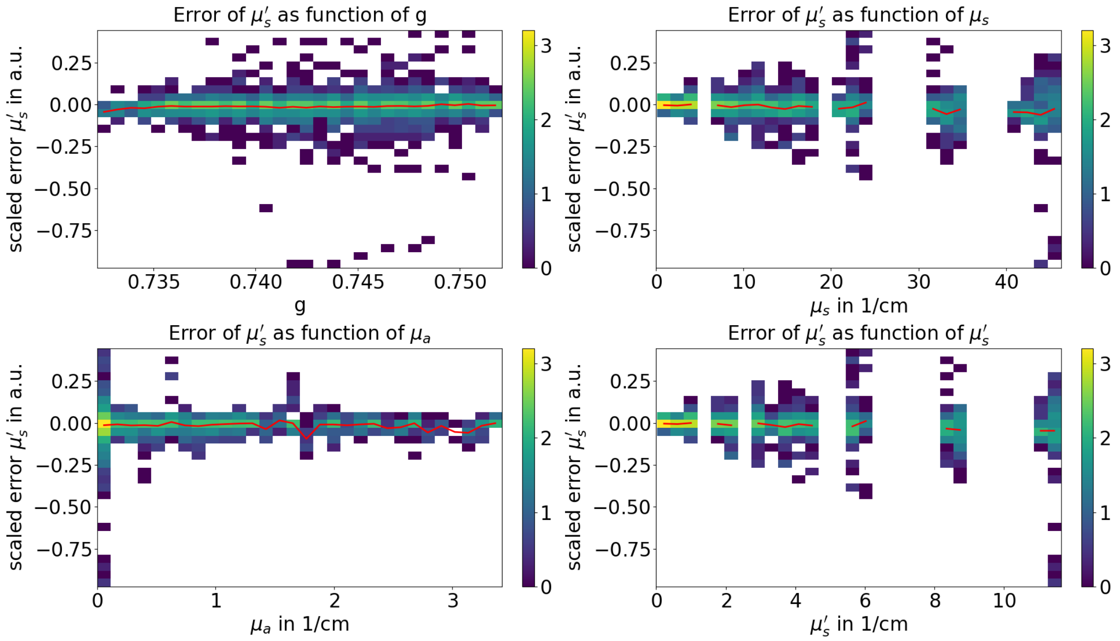

Figure S14: Scaled error of μ_s' as the function of all OPs for Test data: 600 nm $\leq \lambda <$ 630 nm.

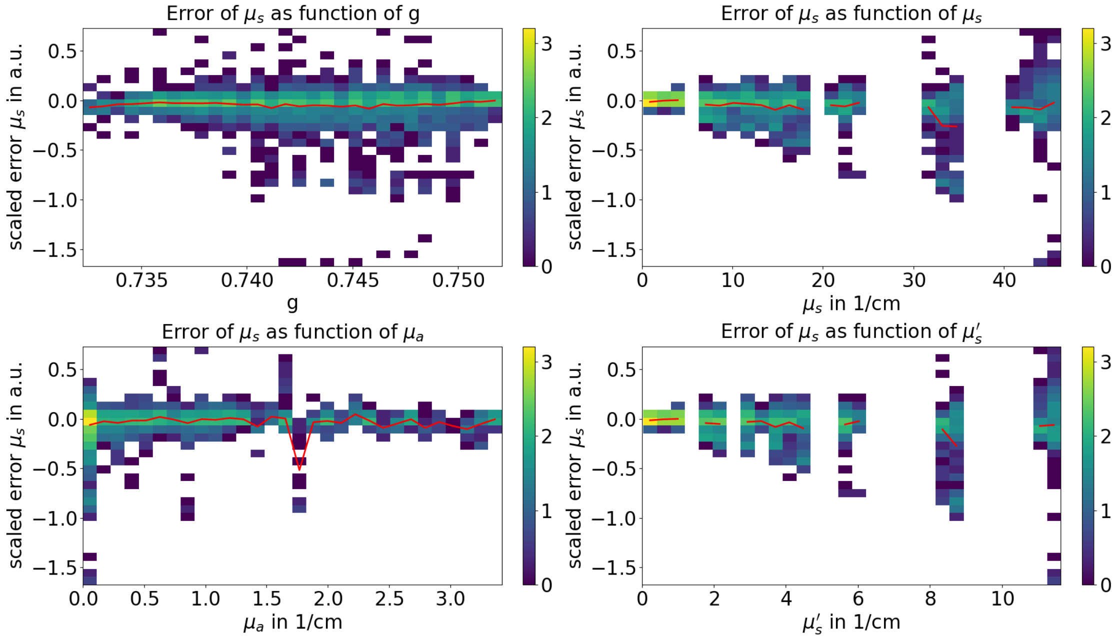

Figure S15: Scaled error of μ_s as the function of all OPs for Test data: 600 nm $\leq \lambda <$ 630 nm.

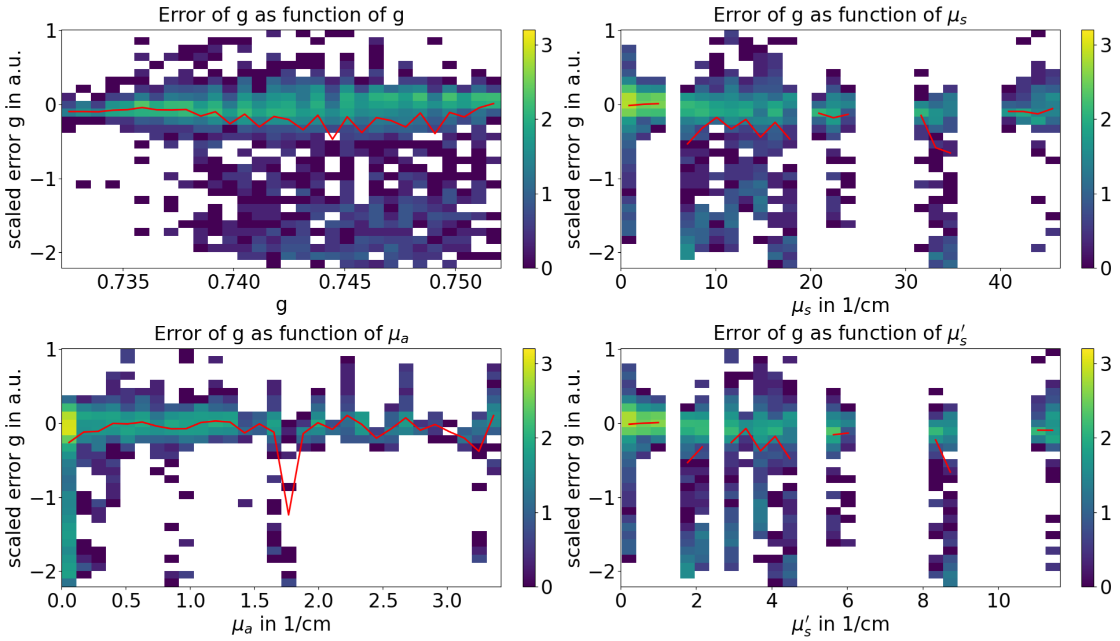

Figure S16: Scaled error of g as the function of all OPs for Test data: 600 nm $\leq \lambda <$ 630 nm.
